# An *in vitro* evaluation of resonant frequency analysis to measure fixed bridge stability

**DOI:** 10.1038/bdjopen.2015.1

**Published:** 2015-10-23

**Authors:** Khaled Omer, Kathryn Fox, Deniel Palermo, Liam Boyle, Callum Youngson

**Affiliations:** 1 Department of Restorative Dentistry, Liverpool Dental School, Liverpool, UK

## Abstract

**Objectives/Aims:**

To determine whether a clinically available resonance frequency analysis (RFA) device (Osstell Mentor), designed to assess implant integration, could identify a single uncemented retainer on fixed–fixed bridges, *in vitro*.

**Materials and Methods:**

All-metal fixed–fixed bridges were cemented to acrylic tooth analogue abutments with simulated periodontal ligaments. Dental stone bases provided 100 or 50% ‘bone support’ groups (*n* = 50 in each). Control groups had both retainers cemented, whereas the test groups had the ‘premolar’ uncemented, mimicking clinical failure. A RFA device was used to measure bridge stability, expressed as a modified Implant Stability Quotient (Bridge Stability Quotient—BSQ) from a Smartpeg temporarily affixed to the bridge via composite.

**Results:**

The BSQ recorded at the premolar site in both 100 and 50% support models demonstrated highly statistically significant differences (*P* <0.003) between the control and test groups. Sensitivity and specificity, area under the curve (receiver operating characteristic), analyses showed moderate test accuracy (0.735) for the 100% support group and good accuracy (0.96) for the 50% support group.

**Conclusion:**

The investigation suggests that RFA measurements were able to identify, reliably and non-destructively, *in vitro*, fixed–fixed bridges where the anterior retainer was uncemented. Further clinical research is required to determine whether this technique may allow early diagnosis of failing bridgework.

## Introduction

Fixed bridge prostheses are a predictable method to replace a missing tooth or teeth and have been reported to exhibit a 10-year survival rate of 72% when placed in the general dental services in the UK.^1^ Bearing in mind the survival rate, this indicates that there is also a substantial failure rate within this period. Loosening of a retainer in a fixed bridge due to luting cement loss is a common clinical complication of fixed prostheses but may be difficult to determine and diagnose. Retainer failure is a challenging situation especially when luting cements fail under a fixed retainer, whereas another retainer remains cemented.^2^ As long ago as 1986, Karlsson^3^ reported that 12.6% of patients had undiagnosed loose fixed prostheses retainers and also noted that a loose retainer occurs more frequently if the abutment had been root canal treated.

Many bridges are affixed to two abutment teeth, one either side of the space in a ‘fixed–fixed’ design. Failure of the luting cement and/or loss of retention on one of these retainers may be disguised by the physical retention offered by the other retainer.^4^ In this case, the prosthesis should be removed in order to prevent recurrent caries of the uncemented abutment and minimise the risk of mechanical damage to the remaining cemented abutment tooth.

The loss of retention, due to failure of the luting cement, often leads to further dissolution of the luting cement and plaque formation. This allows ingress of bacteria that can rapidly cause caries of the underlying tooth. A lack of accessible saliva around the failed retainer means the buffering and remineralising properties of this are absent. Furthermore, the absence of dental enamel (removed during preparation for the bridge) exacerbates the rate of caries.

One investigation has reported that 41% of patients were not aware they had a loose retainer until informed by a dentist.^5^ When patients are aware of a failure, it is usually because they are aware of increased movement developing in the bridge or they start to experience a bad taste from under the loose retainer. By the time a loosened retainer is noticed by the patient, or diagnosed by the dentist, the abutment tooth associated with the failed retainer is often unrestorable.

A method to identify early loss of retention of a fixed bridge would be of a significant benefit. If this could be recognised predictably using a non-invasive method, before caries has taken place, there are several advantages that could be gained. These include minimally invasive removal and recementation of the existing fixed bridge. In addition, early intervention may increase the likelihood of maintaining the retainer with significant biological benefits. Early diagnosis may also preclude the need for a longer-span bridge, which has an increased chance of failure. Replacement of long spans is often not possible with a fixed bridge, and a removable partial denture (with psychological and biological effects) or dental implants, which are associated with morbidity and financial implications, may be the only alternative.

The resonant frequency of an object is a natural frequency at which it can vibrate. The object can filter out other frequencies present in multiple excitations and vibrate at its own resonant frequencies.^6^ In physics, acoustic resonance is when there is an increase in the oscillatory energy of an object in response to another object’s vibration. This oscillation is maximal when the objects both have coincident, or similar, inherent natural frequencies of vibration. The vibration response can be measured and displayed as a frequency reading from which the natural frequencies of the component can be extracted and shown on a screen, which is interpreted as resonance frequency.^7^

In 1996, Meredith *et al.*^8^ described an *in vivo*, non-invasive method of assessing implant stability by measuring the resonance frequency of a small transducer attached to an implant fixture to test the amount of bone formation around an implant. The principle of the method was to attach a transducer either directly to the implant fixture or via a transmucosal abutment using a screw. A further study^9^ tested the practicability of using resonance frequency analysis (RFA) to measure bone height and abutment length *in vivo*, and these authors concluded that resonance frequency measurements are related to the effective length of an implant above the level of the bone. They also mentioned that RFA may be used to monitor changes in stiffness and stability at the implant–tissue interface and could distinguish between successful and failed implants.

**Figure 1:**
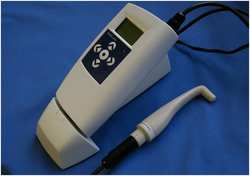
Ostell Mentor.

A commercially available version of a RFA device used in the current investigation is the Osstell Mentor (Figure 1). This device produces magnetic pulses from a probe, which causes a transducer with a magnetic head (Smartpeg), affixed to the implant or abutment by a screw connection, to resonate at high frequency. The Smartpeg is manufactured with a given length and density to have one main resonant frequency in response to multiple excitations from the probe (although other minor frequencies may also be present). The multiple frequencies produced by the vibrating transducer are then analysed by the Osstell Mentor software within the device, and displayed in a simplified form as Implant Stability Quotient (ISQ) values. The software analysis allows the resonant frequencies to be recorded as ISQ values from 0 (very low implant stability) to 100 (high implant stability). Investigations have shown satisfactory repeatability of measurements^10^ and suggested that implants with a primary stability above ISQ 60–65 may be suitable for immediate loading, whereas implants below ISQ 40 may be more prone to failures.^11^ Many studies have investigated the use of the RFA technique to detect clinically stable implants^8,9,12–16^ including those with non-dental uses.^17^

In this current investigation of bridge retainers, rather than implants, values will be expressed in terms of Bridge Stability Quotient (BSQ) instead of ISQ.

### Aims

The primary aim of this investigation is to determine whether an electromagnetic resonant frequency apparatus (Osstell Mentor) is capable of detecting whether, *in vitro*, one retainer of a fixed–fixed bridge is uncemented (a partially cemented bridge).

The secondary aims of this investigation are to:

- Determine whether there is a specific RFA value above which there can be confidence that a bridge is stable *in vitro*.
- Determine an RFA value below which there is a high likelihood of bridge cement failure on one abutment *in vitro.*
- Determine the difference in, *in vitro*, BSQ values between fixed bridges on 100 and 50% simulated periodontal bone support models.

### Null hypotheses

1. There will be no statistically significant differences in BSQ values between fixed bridges in the control and test groups in both the 100 and 50% support groups.
2. There will be no difference in detection, using RFA, of the movement of all-metal bridges, in the ‘uncemented’ and cemented state *in vitro*.

## Materials and methods

A series of pilot studies were conducted to assess the feasibility of testing mobility of bridgework *in vitro* using Osstell Mentor apparatus (Integration Diagnostics AB, Goteborg, Sweden; Figure 1). Smartpegs (Integration Diagnostics AB, Goteborg, Sweden) were temporarily fixed to bridges with composite, and BSQ readings were recorded from various directions to determine the viability and reproducibility of this method. It was observed that these readings were not affected by where the recording was obtained. A standardised, buccal, position of the probe was therefore chosen for subsequent BSQ readings.

In the main investigation, a master model of a standardised fixed–fixed bridge design in the upper left quadrant, from second molar to the second premolar was prepared by one investigator. Wax tooth analogues were then constructed, using the master model as the matrix for the coronal form and appropriate root forms carved for each abutment tooth. Polyvinylsiloxane matrices (Z-Dupe, Henry Schein, Milan, Italy) of these teeth were used to produce wax copies that were subsequently used to produce multiple acrylic analogue (premolar and molar abutment) specimens in heat cured polymethylmethacrylate (Betacryl II, Zhan Laboratory, Henry Schein, Gillingham, UK).

**Figure 2:**
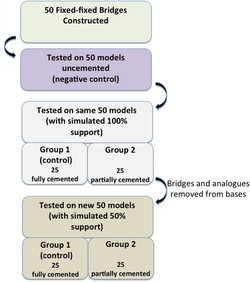
Study design.

Following deflasking and cleaning of the acrylic analogues, the roots to the estimated cement–enamel junction were dipped into polyvinylsiloxane elastomeric impression material to simulate a periodontal ligament. One coat produced a reproducibly acceptable thickness of 0.11–0.14 mm on the ‘root’ surfaces. Working models were then produced by repositioning the acrylic analogues into the silicone index of the originally prepared model and then based with dental stone (Supra-stone, ISO type IV, Kerr, Italy). To determine whether the amount of external support to the root would influence results, 50 models (initially with 100% ‘bone’ support and, subsequently, with 50% support) were produced (Figure 2). Subsequent analysis of the models in the 50% support group demonstrated that the mean root length covered was 50±5%.

**Figure 3:**
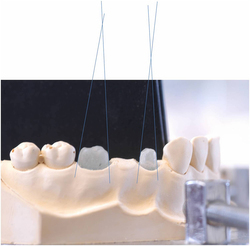
Abutment convergence measurement.

To compensate for any polymerisation shrinkage in the tooth analogues, all analogues had their convergence angles measured. Each abutment was photographed from the buccal and mesial direction using a Pentax K100D DSLR (Ricoh Company Ltd, Tokyo, Japan) fitted with a Tamron 18–200-mm zoom lens (Tamron Co, Saitama City, Japan), tripod mounted, at a fixed distance using ambient light. Using the resultant images, the convergence angles were derived for each abutment (Figure 3). Convergence angles ranged from 12° to 23°. The most frequent were between 14° and 18°. Samples were subsequently allocated in a stratified manner to ensure an even distribution of convergence angles in both the test and control groups.

Fifty standardised, nickel–chrome (Hera, Heraeus Kulzer, GmbH, Hanau, Germany) three-unit bridges were constructed from one master die using a conventional (lost wax) technique with the external contours formed against a silicone index and constructed using conventional laboratory procedures. The casting was tried-in on the working cast and any areas of premature binding were identified using disclosing sprays and adjusted until the casting could be seated passively and removed from the die with gentle finger pressure. Passive fit was confirmed to ensure that no bridge could be retained without cement. Internal surfaces of all castings were finally sandblasted. To act as a negative control all completely uncemented bridges had BSQ values recorded (using the technique detailed later).

Specimens were allocated to test and control groups by a second investigator, who stored the stratification code. This investigator also cemented the bridges using zinc–phosphate luting cement (SS White, Dentsply, UK) with seating pressure applied by an 8 kg weight for 10 min.

After testing of the ‘100% support’ models, to reduce variables, the analogues and bridges were carefully recovered and rebased, by a dental technician who was unaware of the purpose of the investigation, in dental stone to form the 50% support models (Figure 2). In both situations, the bridges were either cemented to both abutments (*n* = 25) or solely to the molar abutment (*n* = 25). Excess cement was removed with a dental probe. Models were sealed inside polythene bags to maintain a constant humidity and prevent the luting cement from dessication before testing.

**Figure 4:**
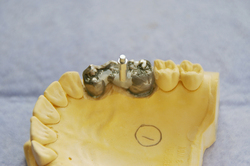
Smartpeg affixed to pontic-molar connector with composite.

Upon removal from the polythene bag, after 7 days, each bridge had a Smartpeg affixed temporarily by light cured composite resin (Synergy D6, Coltene/Whaledent, Burgess Hill, West Sussex, UK) and data recorded at two regions (with removal and repositioning before recording at the second position):

1. At the connector region between the pontic and the posterior retainer (molar) (Figure 4).
2. At the connector between the pontic and the anterior retainer (premolar).

To simulate the clinical situation no attempt was made to completely standardise the length of the Smartpeg above the composite, but this was confined as much as possible to the threaded component (Figure 4) to prevent interference with the resonant frequency of the transducer.

One investigator then recorded BSQ values, with all readings taken from the buccal direction. This investigator was ‘blind’ to which group the models had been assigned and recorded the results by model number. In order to prevent movement during recording measurements, models and associated fixed bridges were placed on a dental surveyor table (model 1451, Nesor Products, London, UK), and fixed with the help of its three stabilising screws. Ten BSQ readings of each bridge were recorded on each occasion and the mean and s.d.’s of these readings were calculated. After all the BSQ values had been recorded, the stratification code was released to assign the data to the control or test groups.

## Results

Initial plotting of data demonstrated that it was not normally distributed therefore descriptive statistics and subsequent non-parametric analysis was used.

### 100% Support group

#### Negative control group (completely uncemented) bridges

The recorded BSQ values for uncemented premolar abutments ranged from 28 to 56 with an average of 39.9±7.3.

Uncemented molar abutment BSQ values ranged from 22 to 57 with an average of 42.3±7.2.

#### Positive control group (cemented–cemented) fixed bridges

BSQ values on the cemented premolar ranged from 43 to 83 with an average of 71.3±10.1.

Cemented molar BSQ values ranged from 44 to 86 with an average of 71.9±9.0.

#### Test group (partially cemented) fixed bridges

BSQ values for the uncemented premolar abutments ranged from 36 to 81 with an average of 58.1±15.0.

The cemented molar abutment BSQ values ranged from 40 to 81 with an average of 66.1±10.2.

Table 1 demonstrates that there is a highly statistically significant difference (*P* <0.005) between the positive control and test groups.

In Table 2, it can be seen that there was a statistically significant difference (*P* <0.05) found between the positive control and test groups.

**Figure 5:**
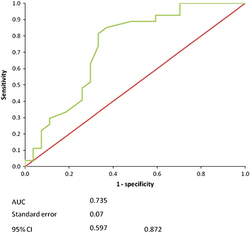
100% support ROC curve.

Table 3 shows the sensitivity (Se), specificity (Sp) and 1−Sp (false-negative ratio) separately. The values of Se start from 1 and decrease as we go further down in the table, whereas Sp decreases as we go up. 1−Sp begins from 1.0 and decreases to reach 0.0 at the bottom of the table. ‘Positive if greater than or equal to’ category is the first column in Table 3, which starts from 35 to end at 87. These numbers represent the BSQ values generated by the receiver operating characteristic (ROC) curve software. The importance of those values is that it allows us to locate the cutoff value for the fixed bridge, above which it is certain the fixed bridge is stable and cemented, or below which it is unstable or moving and may need close observation in the future. From this table, the cross-value to the highest Se plus Sp column is considered to be a cutoff point, which in the present case (in bold text) is 67. The ROC curve with the area under the curve (AUC), s.e. and 95% confidence intervals are shown in Figure 5. The results of ROC curve associated with the premolar readings, in the 100% support study, showed that the AUC was 0.735. This shows moderate test accuracy according to Greiner *et al.*^18^

### 50% support group

#### Positive control group (cemented–cemented) fixed bridges

BSQ values on the cemented premolar ranged from 51 to 85 with an average of 67.8±7.7.

Cemented molar BSQ values ranged from 37 to 84 with an average of 68.6±10.0.

#### Test group (partially cemented) fixed bridges

BSQ values for the uncemented premolar abutments ranged from 41 to 65 with an average of 47.8±6.5.

The cemented molar abutment BSQ values ranged from 32 to 85 with an average of 67.7±10.6.

Table 4 demonstrates that there is a highly statistically significant difference (*P* <0.000) between the positive control and test group at the premolar. However, there was no statistically significant difference (*P*>0.05) between the groups at the molar site (Table 5).

**Figure 6:**
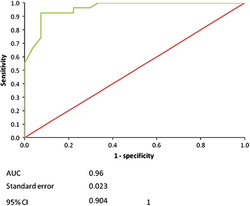
50% support ROC curve.

Table 6 shows the coordinates of the ROC Curve for 50% support. Calculated data start from 40 to end at 86. The cross-value to the highest Se plus Sp is 60 (in bold text) and it is considered to be a cutoff point in the present study. Figure 6 illustrates the ROC curve associated with 50% support, the AUC, the s.e. and 95% confidence intervals. The results of the ROC curve associated with the premolar readings, in the 50% support study, showed that the AUC was 0.96, and this shows high test accuracy.^18^

Analysis of the data suggests that failure was more likely where BSQ values were below 67 in the 100% support models and 60 in the 50% support models.

## Discussion

There are currently only a limited number of ways of detecting clinical bridge failure. On intraoral examination, bubbles of saliva may be detected by pulling/pushing alternately on each retainer of a bridge when complete cement failure has occurred. In addition, the patient may notice a bad odour or taste due to bacterial growth under the retainer which can lead, if not detected in time, to destruction of the tooth structure by caries. This may even be detected radiographically at the margin of bridge retainer of the abutment tooth. However, this approach has its limitations and is usually only an aid to diagnosis at a late stage. In these cases, possible recementation of the fixed bridge is often reduced or impossible. There may be sensitivity to hot or cold with early failure, or severe, spontaneous pain with late (and often unrestorable) failure. An objective method to measure fixed bridge stability, which may enable diagnosis of the early stages of increased movement of these bridges, would therefore be advantageous.

This study aimed to determine whether the novel application of clinically available, non-destructive, RFA would be able to function in this regard. As statistically significant differences in BSQ values were found between fixed bridges in the control and test groups in both the 100 and 50% support models, we can reject the first null hypothesis.

The *in vitro* model chosen had significant limitations. Dental stone bases were used to determine a ‘proof of principle’ but have significantly differing mechanical properties in almost every respect compared with alveolar bone. Similarly, use of a polyvinylsiloxane material has very different characteristics from the visco-elastic properties of the natural periodontal ligament tissues. However, the attempt to create a simulated periodontal ligament to allow some, otherwise indiscernible, movement appears to have been successful. It must be accepted that there may have been some areas where the resultant material thickness could have been very thin, effectively locking the acrylic abutment, and this may have affected the BSQ readings that were obtained. However, every effort was made to ensure the dental stone did not come into contact with the coronal elements of the tooth by covering above the finish line (CEJ) of the acrylic tooth analogues with the elastomeric material. In this way, we intended the abutments to move independently for consistent BSQ readings.

It may be observed that the convergence angles of the analogue abutments were greater than the ideal crown taper suggested by Jorgenson.^19^ However, the aim of the study was to determine whether the loss of cement from the minor retainer could be detected, rather than studying the effect of the retention and resistance form of the abutments and so these preparation tapers were considered reasonable and in line with clinically achieved limits.

The fixation of the Smartpeg was one of the most important variables to control in the present study, using the composite resin to affix it against the metal framework of the bridge at the embrasure areas. The reference point for the placement of the transducer in the same position was that the full length of the Smartpeg serration had to be embedded in composite resin. Both the length and the density of the Smartpeg are very important for the calibration of the instrument, as anything affecting the length may alter the resultant resonance frequency.

In the present study, the sample size was based on our pilot study results, following statistical advice. The diagnostic power of a test is expressed by its Se and Sp of a positive or negative test result. Although a test may be very sensitive, in that no partially uncemented bridges are missed, it may not be specific at all, in that all fixed bridges are stable. There are four possible scenarios in this issue:

1. True-positive decision: the bridge is partially uncemented, is moving, and this has been detected.
2. True-negative decision: the bridge is cemented (no movement) and we state this correctly.
3. False-positive decision: the bridge is stable and we determine that it moves.
4. False-negative decision: the bridge is moving and we state it does not.

In the current study, Se is a measure of bridges moving, as a result of lack of cementation to the anterior abutment, whereas the Sp is a measure of cemented (and thus more stable) bridges. Se is inversely related to Sp and both are used in the ROC curve. Where a decision is required based on uncertain data, ROC can be used to find the agreement in the result. ROC analysis assesses ‘the diagnostic performance of the system in terms of Se and 1-Sp for each possible cutoff value of the test, where Se and Sp are a function of the selected cutoff value’.^18^ There is a potential biological penalty for a false-positive diagnosis, but it may be less harmful in comparison with a false-negative diagnosis (where the bridge is partially uncemented and we state that it is not). Thus, in this case, it is better to accept a poor Sp in order to ensure a very high Se.

The area under the ROC curve in the 100% support group was calculated as 0.735, which presents a moderate accuracy of the resonance frequency test. The interpretation of this result is that: 73.5% of the time a randomly selected sample from the partially uncemented bridge group has a higher chance of being identified than one from the fully cemented group. However, the AUC for the 50% support group should be considered to confer high accuracy, as it is 0.96. This means that the 0.96 point lies in the top left hand corner of the graph and indicates a ‘very or quite confident’ test threshold. ROC analysis provides an estimate of the accuracy that is independent of the specific cutoff value and prevalence.

The actual benefit from a true-positive diagnosis in the present research work is that the potential risk or the serious consequences of a partially uncemented fixed bridge mean that it can be treated in good time, and hopefully help to save the abutment tooth/teeth. The benefit from a false-positive diagnosis, where the bridge is stable and we state it is not, is that any the bridge can be reviewed in future and any future cement failure loss could be diagnosed at an earlier stage.

There is a cutoff point in setting the threshold level and this is to allocate confidence levels to the decisions such as:

1. Very confident that the bridge is partially uncemented.
2. Quite confident that the bridge is partially uncemented.
3. Unsure if the bridge is stable or not.
4. Quite confident that the bridge is cemented.
5. Very confident that the bridge is cemented.

A threshold or cutoff value is required to categorise a test result as positive (abnormal) or negative (normal). Cutoff values are required in test evaluation studies for calculation of Se and Sp and also for clinical decision-making.^20^ The cutoff point from the present study was found to be BSQ at 67 (in 100% support) and 60 (in 50% support). Any respective BSQ value <67 or 60 would lead us to consider a positive diagnosis (i.e., the bridge is loose at one retainer) where only ‘very confident’ or ‘quite confident’ is set at the threshold to be considered a positive diagnosis. Thus, we should have fewer false positives but more false-negative diagnoses. If the results of the present *in vitro* study were directly transferable to the clinical situation, any fixed bridge that has BSQ<60 should be considered as warranting further investigation and would suggest that a focused clinical examination (including radiography) is performed.

Nedir *et al.*^10^ noted that, in immediate-loaded versus delayed-loaded implants, the RFA technique could not identify all mobile implants. The possible cause may have been because the resonance frequency technique measures stability as a function of stiffness, where the mobile implants show low stiffness, which prevents the resonance system from identifying the first resonance frequency. Therefore, it might record a false high resonance frequency quotient in relation to a second resonance frequency.^21^ This explanation may also account for some observations in the present study that the RFA technique could not identify some uncemented bridges. However, to date, there are no other studies that document the role of resonance frequency technique in identifying uncemented bridge in either *in vitro* or *in vivo* studies.

The results of the current study demonstrate that we can reject the second null hypothesis as it has been shown to be possible to use RFA to detect bridge stability differences between partially uncemented and cemented bridges *in vitro*. The results of this *in vitro* study could thus now be used as the basis of an investigation to assess the transferability of the results to the *in vivo* situation.

## Conclusions

- A model that simulates a 50% reduction in periodontal support appeared to increase Se and Sp in the detection of partially uncemented bridges using a RFA device.
- RFA can be used to reliably identify fully cemented bridges and may help to differentiate those that are not cemented to the anterior abutment of a fixed–fixed bridge *in vitro*.
- The cutoff point from the present study was to be found a BSQ value of 67 in 100%, and 60 in 50%, support models.

## Figures and Tables

**Table 1:** Kruskal–Wallis test: 100% support premolar-positive control (1) versus test (2)

*Kruskal–Wallis test (Smartpeg at premolar)*				
*C1*	N	*Median*	*Ave rank*	Z
1	25	72.00	33.8	2.96
2	25	63.00	21.2	−2.96
Overall	50		27.5	

*H* = 8.75, DF = 1, *P* = 0.003.

*H* = 8.77, DF = 1, *P* = 0.003 (adjusted for ties).

**Table 2:** Kruskal–Wallis test: 100% support molar-positive control (1) versus test (2)

*Kruskal–Wallis test (Smartpeg at molar)*				
*C1*	N	*Median*	*Ave rank*	Z
1	25	73.00	32.0	2.09
2	25	67.00	23.0	−2.09
Overall	50		27.5	

*H* = 438, DF = 1, *P* = 0.036.

*H* = 4.40, DF = 1, *P* = 0.036 (adjusted for ties).

**Table 3:** Coordinates of the ROC curve (100% support)

*Test result variable(s): premolar BSQ*				
*Positive if greater than or equal to*	*Sensitivity*	*Specificity*	*1−specificity*	*Sensitivity+specificity*
35.0000	1.000	0.000	1.0	1.000
38.0000	1.000	0.037	1.0	1.037
40.5000	1.000	0.111	0.9	1.111
41.5000	1.000	0.148	0.9	1.148
42.5000	1.000	0.296	0.7	1.296
43.5000	0.926	0.296	0.7	1.222
44.5000	0.926	0.333	0.7	1.259
45.5000	0.926	0.370	0.6	1.296
51.5000	0.926	0.407	0.6	1.333
59.0000	0.889	0.407	0.6	1.296
62.0000	0.889	0.481	0.5	1.370
65.0000	0.889	0.519	0.5	1.407
**67.5000**	**0.852**	0.630	0.4	1.481
68.5000	0.815	0.667	0.3	1.481
69.5000	0.741	0.667	0.3	1.407
70.5000	0.630	0.704	0.3	1.333
71.5000	0.519	0.704	0.3	1.222
72.5000	0.481	0.741	0.3	1.222
73.5000	0.407	0.741	0.3	1.148
74.5000	0.370	0.778	0.2	1.148
75.5000	0.333	0.815	0.2	1.148
76.5000	0.296	0.889	0.1	1.185
77.5000	0.259	0.889	0.1	1.148
78.5000	0.222	0.926	0.1	1.148
79.5000	0.148	0.926	0.1	1.074
80.5000	0.111	0.926	0.1	1.037
81.5000	0.111	0.963	0.0	1.074
82.5000	0.074	0.963	0.0	1.037
83.5000	0.037	0.963	0.0	1.000
85.0000	0.037	1.000	0.0	1.037
87.0000	0.000	1.000	0.0	1.000

**Table 4:** Kruskal–Wallis test: 50% support premolar-positive control (1) versus test (2)

*Kruskal–Wallis test (Smartpeg at premolar)*				
*C1*	N	*Median*	*Ave rank*	Z
1	25	67.00	36.2	5.19
2	25	45.00	14.8	−5.19
Overall	50		25.5	

*H* = 26.94, DF = 1, *P* = 0.000.

*H* = 26.99, DF = 1, *P* = 0.000 (adjusted for ties).

**Table 5:** Kruskal–Wallis test: 50% support molar-positive control (1) versus test (2)

*Kruskal–Wallis test (Smartpeg at molar)*				
*C1*	N	*Median*	*Ave rank*	Z
1	25	70.00	25.8	0.13
2	25	70.00	25.2	−0.13
Overall	50		25.5	

*H* = 0.02, DF = 1, *P* = 0.900.

*H* = 0.02, DF = 1, *P* = 0.899 (adjusted for ties).

**Table 6:** Coordinates of the ROC curve (50% support)

*Test result variable(s):premolar BSQ*				
*Positive if greater than or equal to*	*Sensitivity*	*Specificity*	*1−specificity*	*Sensitivity+specificity*
40.0000	1.000	0.000	1.0	1.000
41.5000	1.000	0.037	1.0	1.037
42.5000	1.000	0.185	0.8	1.185
43.5000	1.000	0.296	0.7	1.296
44.5000	1.000	0.370	0.6	1.370
45.5000	1.000	0.481	0.5	1.481
47.0000	1.000	0.519	0.5	1.519
48.5000	1.000	0.630	0.4	1.630
50.0000	1.000	0.667	0.3	1.667
51.5000	0.963	0.704	0.3	1.667
52.5000	0.963	0.778	0.2	1.741
54.0000	0.926	0.778	0.2	1.704
56.5000	0.926	0.815	0.2	1.741
59.0000	0.926	0.889	0.1	1.815
**60.5000**	**0.926**	0.926	0.1	**1.852**
61.5000	0.889	0.926	0.1	1.815
62.5000	0.852	0.926	0.1	1.778
63.5000	0.815	0.926	0.1	1.741
64.5000	0.741	0.926	0.1	1.667
65.5000	0.667	0.963	0.0	1.630
66.5000	0.556	1.000	0.0	1.556
67.5000	0.481	1.000	0.0	1.481
68.5000	0.407	1.000	0.0	1.407
69.5000	0.370	1.000	0.0	1.370
71.0000	0.259	1.000	0.0	1.259
72.5000	0.222	1.000	0.0	1.222
74.5000	0.148	1.000	0.0	1.148
76.5000	0.111	1.000	0.0	1.111
80.5000	0.074	1.000	0.0	1.074
84.5000	0.037	1.000	0.0	1.037
86.0000	0.000	1.000	0.0	1.000
